# Evaluating Magnetic Stimulation as an Innovative Approach for Treating Dry Eye Disease: An Initial Safety and Efficacy Study

**DOI:** 10.3390/biomedicines13051064

**Published:** 2025-04-28

**Authors:** Hadas Ben-Eli, Shimon Perelman, Denise Wajnsztajn, Abraham Solomon

**Affiliations:** 1 Department of Ophthalmology, Hadassah-Hebrew University Medical Center, Jerusalem 91120, Israel; hadasben@jmc.ac.il (H.B.-E.); shimonperelman@gmail.com (S.P.); denisewaj@gmail.com (D.W.); 2 Department of Optometry and Vision Science, Jerusalem Multidisciplinary College, Jerusalem 91010, Israel

**Keywords:** dry eye, magnetic neurostimulation, cornea, treatment, safety and efficacy

## Abstract

**Objective:** The aim of this study was to assess the safety and preliminary efficacy of repetitive magnetic stimulation (RMS) as a treatment intervention for dry eye disease (DED), focusing on symptom reduction. **Methodology:** This investigation involved 22 adult participants (85% females, aged between 22 and 79 years) diagnosed with moderate-to-severe DED. These individuals were subjected to RMS treatment targeting one or both eyes using the VIVEYE-Ocular Magnetic Neurostimulation System version 1.0 (Epitech-Mag LTD; National Institute of Health (NIH) clinical trials registry #NCT03012698). A placebo-controlled group was also included for comparative analysis, with all subjects being monitored over a three-month period. The evaluation of safety encompassed monitoring changes in best corrected visual acuity, ocular pathology, and the reporting of adverse events. Participant tolerance was gauged through questionnaires, measurements of intraocular pressure (IOP), Schirmer’s test, and vital signs. The efficacy of the treatment was assessed by comparing pre- and post-treatment scores for fluorescein staining (according to National Eye Institute (NEI) grading) and patient-reported outcomes. **Results:** No statistically significant changes were found in visual acuity, IOP, or Schirmer’s test results between the RMS-treated and control groups (*p* < 0.05), indicating that RMS does not negatively impact these ocular functions. However, RMS treatment was associated with improved tear film stability (*p* = 0.19 vs. *p* = 0.04) and corneal health (*p* = 0.52 vs. *p* = 0.004), with no improvements in the control group. Initial symptom improvement was observed in both RMS-treated and placebo groups (*p* = 0.007 vs. *p* = 0.008), suggesting a potential therapeutic benefit of RMS for ocular surface conditions beyond a placebo effect. **Conclusions:** This study presents RMS as a promising therapeutic approach for DED, highlighting its potential to promote corneal epithelial repair, enhance tear film stability, and improve patient-reported symptoms without negatively impacting IOP, visual acuity, or tear production. This confirms the safety and suggests the efficacy of RMS therapy for dry eye conditions.

## 1. Introduction

Dry eye disease (DED), also referred to as keratoconjunctivitis sicca (KCS), is identified by the International Dry Eye Workshop (DEWS, 2007) [1] as a multifactorial disorder impacting the tears and ocular surface. This chronic condition leads to discomfort, visual disturbances, an unstable tear film, and possible ocular surface damage [2]. DED is a common diagnosis in ophthalmology, with a growing prevalence ranging from 5 to 33% in the adult population worldwide [2,3,4] and even higher, up to 87%, in visual display terminal workers [5]. Notably, 78% of patients with DED are women [3]. The symptoms, including irritation, stinging, and fluctuating visual disturbances, can progress to severe complications such as vision impairment and corneal damage if left untreated [6,7].

DED is influenced by both intrinsic (e.g., autoimmune disease, hormonal changes, aging) and extrinsic (e.g., environmental pollutants, prolonged screen use) factors. It is assumed that all these etiological and risk factors are associated with the disruption of the structure or function of one or more of the tear film layers, ultimately leading to corneo-conjunctival epithelial damage. This disruption contributes to ocular surface inflammation and neurosensory dysfunction, further exacerbating disease severity [2,3]. The cornea, one of the most highly innervated tissues, contains superficial nerve endings in direct contact with the environment. A variety of external stimuli can activate the trigeminal pathway, transmitting signals to the somatosensory cortex and limbic system, leading to nociception [4].

Traditional treatments for DED largely involve topical lubricants and anti-inflammatory drops. Despite their widespread use, these methods pose economic challenges and often demonstrate limited efficacy, particularly in severe cases requiring long-term management [4,8]. Recent advancements have introduced treatments based on heating and massaging the eyelids and Meibomian glands, but these can be painful and provide only short-term relief [9,10,11]. In parallel, pharmacological innovations have introduced novel treatments such as long-acting biodegradable inserts (e.g., Lacrisert) for sustained lubrication [5] and lipid-based sprays to stabilize the tear film and reduce evaporation [5]. Emerging agents such as naltrexone and tacrolimus show promise for their anti-inflammatory effects, with naltrexone reducing ocular surface inflammation [7] and tacrolimus improving tear production [12]. Additionally, neuromodulatory approaches such as repetitive magnetic stimulation (RMS) are being explored for their role in enhancing tear production by modulating neural pathways [13]. These innovations complement existing treatments, including cyclosporine (RESTASIS^®^, VEVYE^®^, CEQUA™), lifitegrast (XIIDRA^®^), loteprednol etabonate (EYSUVIS™), and perfluorohexyloctane (MIEBO™), offering new strategies to manage DED more effectively [5,6]. While these therapeutic advancements show promise, most interventions primarily target symptom relief, inflammation, or tear film stability, often requiring frequent application or long-term use. RMS, in contrast, presents a unique, non-invasive approach that may directly influence corneal nerve function and tear production pathways. Unlike conventional pharmacological treatments, RMS is designed to modulate neural activity, potentially offering longer-lasting effects with minimal patient burden. However, limitations such as the optimal treatment frequency, the durability of effects, and the need for further large-scale validation remain areas for future investigation.

A novel non-invasive treatment based on RMS was recently studied in a preclinical trial and was reported to be highly effective in protecting the corneal epithelium in a rabbit model of short-term exposure keratopathy. RMS treatment led to a marked reduction in epithelial defects, as indicated by decreased fluorescein staining scores and improved epithelial cell morphology. Histological analysis further demonstrated enhanced corneal healing, likely mediated by the neuromodulation-induced secretion of trophic factors and neuropeptides involved in epithelial regeneration. These findings suggest that RMS may facilitate corneal repair by stimulating sensory nerve endings, promoting cellular proliferation, and accelerating wound healing [14]. Based on these preliminary findings, our group conducted a first-in-human clinical trial to evaluate this treatment in patients with DED. This study represents the first clinical application of RMS for DED. Neuromodulation, a novel therapeutic approach, utilizes electrical signals to modulate abnormal neural function through targeted neurostimulation. This newly developed technique specifically stimulates corneal nerve endings via electrical currents, which modulate tear secretion and epithelial healing through neurosensory mechanisms. RMS operates through either an external electric field [14] or intranasal neurostimulation therapy to enhance tear production [7,8].

The RMS treatment was developed based on the transcranial magnetic stimulation (TMS) approach, which is based on neurostimulation and neuromodulation and is in clinical use (Food and Drug Administration (FDA)-approved since 2008) for the treatment of a variety of neurological and psychiatric disorders such as obsessive–compulsive disorder (OCD) [10], depressive disorders [11], schizophrenia, and Parkinson’s disease [15]. Beyond its use in psychiatric disorders, repetitive peripheral magnetic stimulation (rPMS) has been explored for neuromodulation in various medical conditions, including peripheral nerve disorders. Savulescu et al. (2021) demonstrated therapeutic effects of rPMS in lumbar radiculopathy, suggesting its potential to modulate peripheral nerve activity [16]. This supports the hypothesis that RMS may similarly influence corneal nerve function to improve dry eye symptoms. According to the International Neuromodulation Society, neuromodulation refers to the targeted alteration of nerve activity using electrical or chemical stimuli [17].

Repetitive transcranial magnetic stimulation (rTMS) is thought to modulate neuronal systems through various mechanisms. These include altering neurotransmitter and ion channel activities, inducing intra-cortical inhibition and long-term potentiation, and affecting gene expression and growth factor production. It also impacts signaling pathways and the glutamate-mediated blood–brain barrier. Additionally, rTMS is believed to stimulate parasympathetic innervation to the lacrimal glands [9,10].

The current study focuses on the application of RMS in humans, following its efficacy in decreasing epithelial corneal erosions in a rabbit model of exposure keratopathy [4]. In this study, we assessed the safety and efficacy of a novel non-invasive instrument designed for treating patients with DED, marking the first human trial of the RMS procedure.

## 2. Materials and Methods

### 2.1. Study Design

This study was a prospective, hospital-based, interventional, open-label, and single-group-assignment study (#ClinicalTrials.gov identifier: NCT03012698) [18].

#### 2.1.1. Study Aims

The primary objective of this study was to evaluate the safety of RMS as a treatment for DED. The secondary objectives were to assess the tolerability of the treatment and to determine its preliminary efficacy in reducing signs and symptoms of dry eye.

#### 2.1.2. Study Endpoints

The primary endpoint of this study was the evaluation of successful RMS treatment for DED measured by a lack of deterioration in the best corrected visual acuity (BCVA) and a reduction in DED signs and symptoms. The secondary endpoints were based on safety and tolerability measures: pathological ocular changes observed in a slit lamp biomicroscopy assessment, any adverse events, questionnaire-based tolerability assessment, intraocular pressure (IOP), Schirmer’s test, and vital signs (heart rate, blood pressure, and body temperature) were all indications for trial termination. Efficacy secondary endpoints included clinically and statistically significant reduced fluorescein staining scores between baseline and post-treatment visits at any of the follow-up time points, reduced ocular discomfort between baseline and post-treatment visits at any of the follow-up time points (questionnaire score), and an improvement in the tear film as measured in tear break-up time (TBUT).

### 2.2. Participants

This study included 22 male and female adult patients (85% females), aged between 22 and 79 years, with moderate-to-severe DED classified by severity of signs and symptoms [17] with different etiologies (meibomian gland dysfunction (MGD), Sjögren’s syndrome (SS), aqueous tear deficiency (ATD), and graft-versus-host disease (GVHD)), recruited at the Ophthalmology Clinics of Hadassah Medical Center. Patients received one-time treatment with the VIVEYE-Ocular Magnetic Neurostimulation System version 1.0 (Epitech-Mag LTD., Yokneam, Israel, 2016). Each participant underwent an initial RMS treatment session with the VIVEYE. In a subset of participants, additional sessions were administered as part of an extended protocol.

The follow-up period lasted 12 weeks and included 10 evaluations (screening, baseline, treatment, day 1, months 1, 2, and 3, along with biweekly phone calls). At each visit, patients underwent assessments for treatment safety, efficacy, and symptom severity. Participants who wore contact lenses were instructed to discontinue use for the study duration.

This study was approved by the national Ministry of Health (#20162621) and by the institutional Helsinki committees of Hadassah Medical Center (#HMO-0630-16; #HMO-0405-19). This study was also registered in the National Institute of Health (NIH) clinical trials registry (#ClinicalTrials.gov identifier: NCT03012698 [18]). Recruitment started on 4 December 2017 and ended on 25 April 2021. All participants provided written informed consent after receiving verbal and written explanations of the study. Data were anonymized for analysis.

#### 2.2.1. Inclusion and Exclusion Criteria

The study included male and female participants aged 22–79 years diagnosed with moderate-to-severe DED who met the eligibility criteria and provided informed consent. Individuals with other ocular surface pathologies requiring treatment beyond ocular lubricants and conventional eyelid hygiene, concurrent ocular diseases such as ocular infection or pterygium, recent ocular surgery (within the preceding 6 months) or laser-assisted in situ keratomileusis (LASIK) (within the year prior to recruitment), ocular injury, or ocular herpes infection within the preceding 3 months were excluded. Also, patients who had recently taken central nervous system drugs, required contact lenses during the study, had thyroid disorders or alcoholism, were pregnant or nursing, had human immunodeficiency virus (HIV), or had various implants such as pacemakers or cochlear implants were not included in this study. Additionally, patients with significant heart or brain diseases, patients with a history of neurological conditions, or those who had recently participated in another ophthalmic trial were also excluded.

#### 2.2.2. Instrument

**Figure 1:**
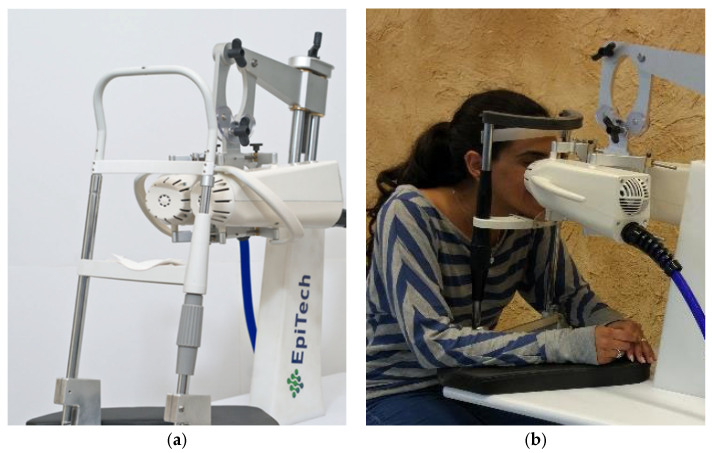
The VIVEYE-Ocular Magnetic Neurostimulation System version 1.0 (Epitech-Mag LTD), a non-invasive device designed for localized electromagnetic stimulation of the cornea in adult patients with DED. (**a**) Overview of the VIVEYE system, including the main control unit and stimulation coil; (**b**) A patient undergoing treatment with the device, with the stimulation coil positioned near the eye to deliver targeted magnetic stimulation.

The VIVEYE-Ocular Magnetic Neurostimulation System version 1.0 (Epitech-Mag LTD.) is a non-invasive stimulation device intended for the application of localized electromagnetic stimulation to the cornea in adult patients with DED (Figure 1) (device detailed overview in Supplementary Figure S1). Its main components are a Rapid2 stimulator unit and a pair of coil applicators. The applicators are attached to a positioning device for the adjustment of their position relative to the patient’s eyes. Only one coil is presented in the stimulator at a time, allowing the treatment of only one eye per session. The system uses a commercial ophthalmic table (Conformité Européenne (CE)-marked) and chin rest to adjust to various patient sizes. The Rapid2 stimulator is the central component of the system and controls the various properties of the magnetic stimulation, such as intensity and rate. It consists of a generator unit, a touchscreen for selecting the treatment parameters and triggering the stimulation, and a set of coils. This device complies with international harmonized standards for the clinical investigation of medical devices (ISO 14155: Clinical investigation of medical devices for human subjects).

Each eye was treated separately in the VIVEYE-Ocular Magnetic Neurostimulation System, taking about 11 min to complete a set of magnetic pulses. Each participant received demo magnetic pulses on the hand and the eye prior to the treatment to understand the sensation of the light vibration stimulated by the device. Any metallic objects around the face were removed before treatment. Magnetic stimulation was delivered at a fixed frequency of 20 Hz. The intensity was set to a maximum of 50% of the device’s output, adjusted lower if necessary to accommodate patient tolerance. Frequency-specific efficacy was not assessed as the study focused on this fixed stimulation parameter.

#### 2.2.3. Safety and Efficacy Tests

Safety and efficacy tests of the VIVEYE-Ocular Magnetic Neurostimulation System were based on ophthalmic and vital signs that were evaluated at each follow-up visit. Safety tests included the assessment of treatment-related adverse or serious adverse events, best corrected visual acuity (BCVA, Early Treatment Diabetic Retinopathy Study (ETDRS) chart, Logarithm of the Minimum Angle of Resolution (LogMAR) units), IOP measurement, Schirmer’s II test (with local anesthesia, measuring mm/5 min) [19], external eye examination by slit lamp biomicroscopy assessment, fundus examination (with dilation at baseline visit, 1 day, 1 week, and 12 weeks post treatment), and Spectral-Domain Optical Coherence Tomography (SD-OCT) [20] at the baseline visit and 1 and 12 weeks post treatment.

Treatment efficacy tests included TBUT [1,21], which was repeated three times, with the mean result recorded, and corneal fluorescein staining photography (Slit Lamp, BI 900, Haag-Streit Group, Köniz, Schweiz), in which two strips of fluorescein were diluted in 500 µL of saline solution for 1.5 min, and then 0.2 μL was inserted into the conjunctival fornix. Subjective grading of the corneal erosion and fluorescein staining was conducted using the well-validated National Eye Institute (NEI) grading scale, commonly used in clinical settings [22,23]. The NEI scale divides the cornea into five different areas, and each area is given a subjective score between 0 and 3 based on the number, size, and confluence of the superficial punctate erosions. To comprehensively assess the efficacy of this novel DED treatment, we implemented a multidimensional evaluation to quantify its impact on both symptoms and quality of life. For this purpose, we selected two distinct questionnaires, each with a unique focus and strength, to ensure a comprehensive evaluation. At each visit, the patients answered quality of life (QoL) and eye dryness symptom questionnaires (modified standard patient evaluation of eye dryness (SPEED) questionnaire [24] and patient-reported outcomes with laser in situ keratomileusis (PROWL) questionnaire [25]), and the visual analog scale (VAS) questionnaire for eye dryness monitoring [26]. QoL surveys were assessed for each participant throughout the three-month follow-up duration.

To normalize the outcomes, a scoring system was applied wherein the minimum QoL scores were allocated a value of 1 and the maximum scores were given a value of 5. This method of scoring was crucial for integrating the results from the different QoL questionnaires used in our study, allowing for a unified analysis of the data.

#### 2.2.4. The Course of the Experiment

Patients presenting to the cornea clinic at the Hadassah Medical Center with dry eye symptoms were invited to participate. Eligible participants underwent screening to confirm inclusion criteria and assess dry eye severity using a validated symptom and sign grading scale [19]. Only patients with moderate-to-severe levels of eye dryness were included in the current study. The severity grading and all the ophthalmic clinical evaluations were carried out by cornea specialists (A.S., D.W.). A detailed systemic and ophthalmic history was documented for each participant.

The follow-up period was 12 weeks and included 8 visits: screening, baseline, treatment, and 1 day, 1 week, 4 weeks, 8 weeks, and 12 weeks post-treatment, as well as biweekly phone calls at 2, 6, and 10 weeks (see the study visit scheme in Supplementary Figure S2). Each follow-up by phone included an eye dryness questionnaire and drug intake monitoring. Follow-up visits in the clinic also included many safety and efficacy tests, as detailed above. The study was conducted in two phases. In the first phase, treatment was applied to one eye per patient, while the contralateral eye remained untreated (N = 7). Participants were blinded to which eye received treatment. Although both eyes underwent identical procedures, only one eye received magnetic stimulation, while the other eye received a placebo treatment. In the following phase, the participants were divided into two groups: one received treatment for both eyes during the same session (N = 9), whereas the other group received placebo treatments for both eyes (N = 6). Two participants did not complete all scheduled follow-up visits and were excluded from statistical analysis.

#### 2.2.5. Statistical Analysis

Quantitative variables are presented as the mean and standard deviation, and changes across different time points were assessed using the Friedman nonparametric test. Comparison between quantitative variables for two independent groups was conducted using either the *t*-test or the nonparametric Mann–Whitney U test. The simultaneous evaluation of time, treatment effects, and their interaction was achieved through the application of the repeated-measures analysis of variance (ANOVA) model, employing the Greenhouse–Geisser test. Associations between two categorical variables were tested using the Chi-square test and Fisher’s exact test. The utilization of nonparametric tests was prompted by the limited sample size. All statistical tests were two-tailed, with a significance threshold set at *p* value ≤ 0.05 to determine statistical significance. Statistical analysis was performed using JMP^®^ Statistical Discovery software, version 14.3.0, from SAS^®^ Institute Inc. (Cary, NC, USA).

## 3. Results

This study included 20 patients, with a mean age (±standard deviation (SD)) of 51.4 ± 18.6 years in the treated group and 47.7 ± 17.9 years in the non-treated group. The overall age range was 22–79 years (Table 1). As expected, most of the study participants were women in both groups (N = 16; 84% and N = 22; 88%, respectively). SS was the most common clinical manifestation among the treated and non-treated groups. Other common etiologies were aqueous tear deficiency and meibomian gland disfunction. While the treatment groups were composed mostly of these three etiologies, there were no statistically significant differences in the dispersion of different etiologies between groups (Table 1). Baseline measurements of IOP, BCVA, and Schirmer’s test showed no significant differences between the two groups. There were no statistically significant differences in baseline IOP, BCVA, or tear production between the treated and control groups (Table 1).

RMS safety was evaluated using multiple assessments, including BCVA measurements at different time points for both treated and non-treated eyes (Table 2). The BCVA measurements were obtained for both treated and non-treated eyes at enrollment and subsequently at 1 week, and 1, 2, and 3 months. The mean BCVA values demonstrated no statistically significant differences between the treated and non-treated eyes across different time points, as shown in Table 2.

To further assess the safety of RMS, we compared IOP values over time between treated and non-treated groups. The IOP measurements at baseline (enrollment) and subsequent time points (1 week, 1, 2, and 3 months) are presented in Table 2. Overall, while both groups exhibited decreases in mean IOP over the 3-month period, both treated groups did not show consistent reduction. Nevertheless, both groups exhibited stability in IOP and showed no statistically significant changes over time, as shown in Table 2.

To further assess the safety of RMS treatment, we monitored changes in Schirmer’s test values over a 3-month period in two distinct groups, the treated and non-treated cohorts. The mean and standard deviation values for each group are presented in Table 2. In the non-treated group, despite observable fluctuations, the change over time did not achieve statistical significance (*p* = 0.51). Similarly, the change over time within the treated group did not show statistical significance (*p* = 0.41).

When comparing the change over time between the two groups, no statistically significant difference emerged (*p* > 0.05). Both the non-treated and treated groups displayed variable Schirmer’s test values over the study period but with no statistically significant change between the groups.

Efficacy was assessed over three months by measuring NEI scores at multiple time points. We evaluated the total scores across different time points for both the treated and non-treated groups. Table 3 provides a summary of the mean NEI total scores along with corresponding sample sizes and the results of statistical comparisons.

The efficacy of RMS was also assessed by TBUT changes over a three-month period in the two groups, treated and non-treated (Table 3).

**Figure 2:**
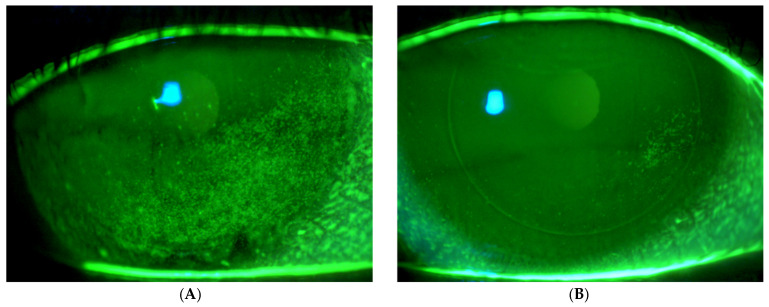
Fluorescein images of the treated eye of a 46-year-old male with Sjögren’s syndrome. (**A**) Pre-treatment images show multiple corneal erosions (NEI score 8), while (**B**) post-treatment (8 weeks) images reveal significant improvement with fewer erosions (NEI score 2) following VIVEYE Neurostimulation System therapy.

Figure 2 presents fluorescein imaging of the treated eye of a 46-year-old male patient diagnosed with SS and severe eye dryness. The patient had undergone LASIK surgery in 2012, six years prior to the SS diagnosis and seven years before enrollment in the current study involving treatment with the VIVEYE Neurostimulation System. The images reveal multiple superficial corneal erosions before treatment, with an NEI score of 8. Following eight weeks of treatment, the same eye exhibited significantly fewer epithelial erosions, with an improved NEI score of 2.

**Figure 3:**
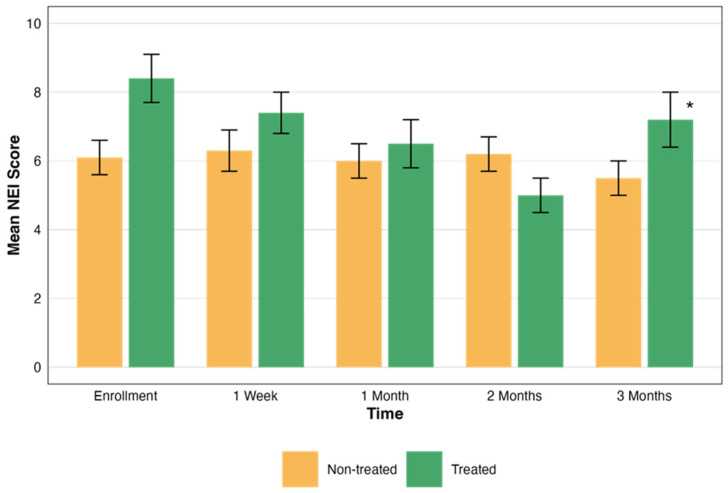
Comparative assessment of NEI scores over time. This graph illustrates the changes in the NEI total scores over a period of three months, comparing groups that were treated (green) and not treated (orange). Each bar represents the mean ± standard error (SE) NEI total score at five time points: enrollment, 1 week, and 1, 2, and 3 months. The asterisks represent the statistical significance of the differences observed (*p* < 0.05).

In the non-treated eye group, the mean NEI scores did not show significant changes over time. Conversely, the treated group demonstrated a statistically significant improvement. The statistical significance of these changes was evaluated, revealing a significant difference in the treated group’s NEI total scores over time (*p* = 0.004). However, the NEI total scores in the non-treated group did not show significant changes over time (*p* > 0.05). These scores are presented in Figure 3.

**Figure 4:**
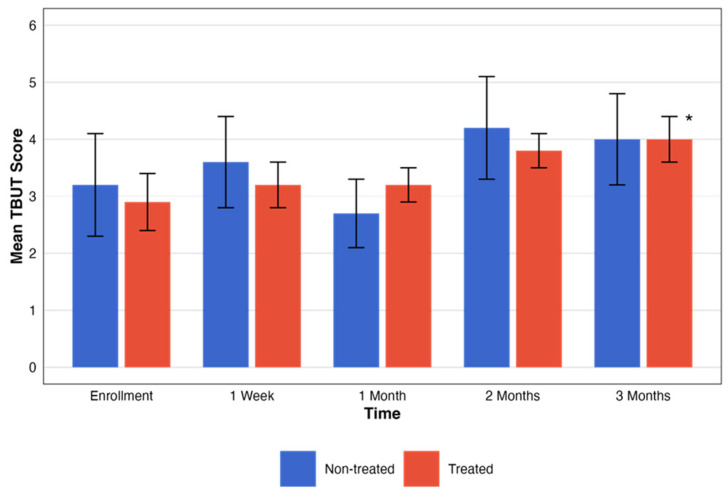
Comparative assessment of TBUT between treatment groups. This figure illustrates the temporal progression of TBUT scores across five different time points. The graph compares groups that were treated (red) and not treated (blue). Each bar represents the mean ± SE TBUT score. The black asterisks represent the statistical significance of the differences observed (*p* < 0.05).

For the non-treated group, no statistically significant changes in TBUT scores were observed across the designated time intervals (*p* = 0.19). Contrarily, TBUT scores for the treated group showed an improvement in the period tested with statistical significance (*p* = 0.04). These changes, with the pattern of change in the testing period, are presented in Figure 4.

In addition, an evaluation of the subjective assessment scores was performed for both the non-treated and treated groups. Over the corresponding 3-month period and matching time points, the subjective score for each patient was measured (Table 4). Both the treated and placebo groups showed statistically significant improvement in subjective symptom scores (*p* = 0.007 vs. *p* = 0.008, respectively). However, the difference between groups was not statistically significant (*p* = 0.09), suggesting a potential placebo effect. Despite this, objective parameters such as fluorescein staining and TBUT were significantly improved only in the treated group (*p* = 0.004 and *p* = 0.04, respectively), supporting a potential physiological benefit of RMS.

**Figure 5:**
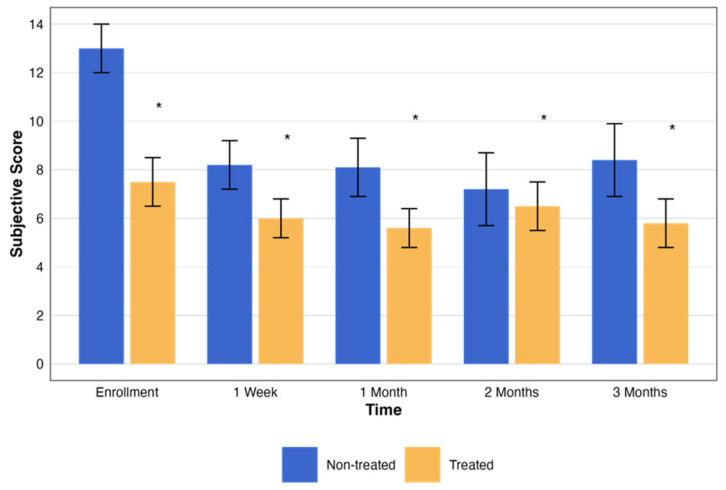
Change in subjective assessment score over time by treatment group. This graph illustrates the changes in the NEI total scores over a period of three months, comparing groups that were treated (orange) and not treated (blue). Each bar represents the mean ± standard error (SE) NEI total score at five time points: enrollment, 1 week, and 1, 2, and 3 months. The asterisks represent the statistical significance of the differences observed (*p* < 0.05).

The subjective assessment scores exhibited notable changes throughout the study. Both the treated and non-treated groups had statistically significant changes (*p* < 0.001 and *p* < 0.005, respectively). These changes, with the pattern of change in the testing period, are presented in Figure 5.

## 4. Discussion

This study investigated the safety and preliminary efficacy of repetitive magnetic stimulation (RMS or rTMS) as a first-in-human therapeutic intervention for DED. It included 22 adult subjects diagnosed with moderate-to-severe DED who underwent treatment utilizing the VIVEYE-Ocular Magnetic Neurostimulation System version 1.0. The primary endpoints of this study were safety, tolerability, and preliminary effectiveness. Our findings indicate that RMS is a safe and well-tolerated modality, resulting in the significant amelioration of dry eye symptoms, as evidenced by improved fluorescein staining scores and reduced patient-reported ocular discomfort, underscoring the innovative potential of this treatment in managing DED.

Recent studies have highlighted the growing interest in non-invasive neuromodulation techniques for ocular diseases, particularly in the management of DED. Trigeminal nerve stimulation (TNS) has been shown to improve tear production and ocular surface health. For instance, intranasal neurostimulation has been demonstrated to increase tear volume and alleviate symptoms of dryness and ocular pain [27,28].

RMS is another emerging modality in this field. Although traditionally used for neurological conditions, RMS has shown potential in modulating neural pathways involved in tear production and ocular surface health. The International Federation of Clinical Neurophysiology has reviewed non-invasive brain stimulation techniques, including RMS, for various ocular conditions, suggesting their potential efficacy in enhancing tear production and managing DED [29]. These findings support the role of neurostimulation therapies, including TNS and RMS, as promising interventions for DED. However, further research is needed to establish their long-term efficacy and optimize treatment protocols.

Neurostimulation, as indicated in prior animal research, influences epithelial cells via the activation of trigeminal nerve endings [14,30,31]. The proposed mechanism involves the secretion of neurotransmitters and neurotrophic factors [14], although the precise pathways remain unclear. RMS may exert therapeutic effects by modulating the parasympathetic innervation of the lacrimal gland and conjunctival goblet cells, thereby enhancing tear production and improving ocular surface health [32].

Moreover, repetitive transcranial magnetic stimulation has been linked to long-term biological changes, including alterations in gene and protein expression, suggesting sustained neuromodulatory effects [33,34,35,36]. One proposed mechanism involves substance P, a trigeminal neuropeptide that has been observed to promote cell attachment through E-cadherin and stimulate DNA synthesis and growth in vitro [37,38], ultimately leading to epithelial cell proliferation [39]. Collectively, these findings suggest that RMS may provide corneal protection in animal models, positioning it as a potentially safe and effective therapeutic option for patients with exposure keratopathy [14].

Various neurostimulation modalities have been explored for the treatment of DED, including transcutaneous electrical nerve stimulation (TENS), intranasal tear stimulation (ITS), and lacrimal nerve stimulation (LNS). While all these approaches aim to enhance tear production via neuromodulatory mechanisms, they differ significantly in terms of treatment duration, invasiveness, patient compliance, and efficacy. TENS applies electrical stimulation to the periorbital region and has been effective in increasing tear production and alleviating dry eye symptoms. However, it requires multiple sessions per week, which may reduce patient adherence. Additionally, the discomfort associated with prolonged external electrode placement may limit its long-term use [40]. ITS stimulates the nasal mucosa to trigger tear secretion and has demonstrated clinical benefits in DED. However, its direct nasal insertion may be uncomfortable for some patients, requiring frequent daily applications for sustained efficacy [41]. LNS, a more invasive method, directly stimulates the lacrimal nerve to enhance tear secretion. While promising, it requires surgical implantation, making it a less favorable option due to procedural risks and long-term maintenance requirements [42].

Compared to these modalities, RMS offers distinct advantages. It is entirely non-invasive, requiring no surgical procedures or continuous patient involvement. Unlike TENS and ITS, RMS does not necessitate frequent daily applications. In our study, a limited number of sessions provided measurable clinical benefits. Additionally, RMS has demonstrated potential effects beyond tear stimulation, including corneal epithelial protection and neuromodulation, which may contribute to long-term improvements in ocular surface health. These characteristics suggest that RMS may be a more practical and patient-friendly approach for DED management.

Unlike previous studies, our investigation highlights distinct advantages that may enhance treatment outcomes and patient adherence. For instance, while TENS studies have required twenty treatment sessions, with over five sessions per week [29], our protocol involved only four sessions. Furthermore, unlike ITS, which involves nasal application and may cause discomfort (e.g., epistaxis) [31], RMS is non-invasive, eliminating procedural discomfort. These distinctions underscore the benefits of RMS over prior methods, offering a non-invasive and more manageable treatment approach. Consequently, these characteristics are likely to enhance patient adherence and overall satisfaction, underscoring the potential for improved clinical outcomes and patient experiences.

The current study has several limitations that may affect the generalizability of its findings. First, the relatively small sample size, largely due to the high costs associated with conducting the trial, may limit the applicability of the results to a broader population. Second, the study cohort consisted exclusively of individuals with moderate-to-severe DED, meaning that the observed benefits of repetitive magnetic stimulation (RMS) may not extend to those with milder forms of the condition. Third, the open-label study design introduces potential bias in patient-reported outcomes, as some participants were assigned to a placebo group while others received active treatment in both eyes. Although efforts were made to mitigate this through the inclusion of a placebo group and objective measures, subjective assessment variability remains a challenge.

Additionally, while this study primarily assessed the effects of a single RMS session, a subset of participants underwent four sessions in a secondary phase. This raises important questions about treatment frequency and long-term efficacy. Future research should evaluate the impact of multiple treatment sessions to determine the optimal regimen for symptom persistence and therapeutic benefit. Given these methodological constraints, a double-blind, randomized controlled trial is necessary to confirm our findings and reduce potential confounding effects related to placebo response and subjectivity in symptom reporting.

Despite these limitations, our study demonstrated significant improvements in both the signs and symptoms of DED, reinforcing the potential of RMS as a novel therapeutic intervention. Our findings suggest that RMS promotes corneal epithelial healing, enhances tear film stability, and alleviates ocular discomfort, with a favorable safety and tolerability profile.

This study introduces a non-invasive, cost-effective, and efficient therapeutic approach for managing DED. To our knowledge, this is the first clinical investigation of RMS for DED, expanding its application beyond its established use in psychiatric conditions, such as OCD, where frequent treatment sessions are typically required. Our protocol implemented a single-session RMS treatment, demonstrating promising outcomes. This initial evidence supports the further exploration of RMS, including adjustments to treatment frequency, stimulus parameters, and extended session protocols, which may enhance therapeutic efficacy.

Preclinical findings further support the potential benefits of RMS. Animal studies, particularly in rabbit models, have demonstrated its efficacy in corneal protection and epithelial regeneration, suggesting that it may serve as a safe and effective treatment for exposure keratopathy. The non-invasive and pain-free nature of RMS, coupled with its rapid therapeutic effects, positions it as a viable alternative for patients unresponsive to conventional therapies. The need for infrequent treatment sessions—spaced weeks or months apart—further enhances its cost-effectiveness and patient convenience, potentially reducing reliance on daily eye drop regimens.

Moreover, the simple application process of RMS allows for its use across various healthcare settings. Its ease of administration makes it accessible not only to ophthalmologists and optometrists but also to trained office staff and nursing personnel, broadening its clinical applicability. While these findings are encouraging, further research is essential to validate these results, establish long-term efficacy, and determine the most effective treatment parameters. Future studies should explore longitudinal effects, the optimal treatment frequency, and comparative effectiveness against existing DED treatments to solidify the role of RMS in clinical practice.

## 5. Conclusions

This study presents repetitive magnetic stimulation (RMS) as a novel, non-invasive therapeutic approach for DED. Our findings highlight the safety and potential efficacy of RMS in stabilizing the tear film, reducing corneal damage, and improving patient-reported symptoms, without adversely affecting IOP, visual acuity, or tear production. These results support the integration of RMS into the therapeutic landscape for DED, marking a potential shift toward non-pharmacological treatment strategies.

## Figures and Tables

**Table 1:** Basic characteristics of study participants.

Characteristic	Treated Eye (N = 21) Mean ± SD	Non-Treated Eye (N = 19) Mean ± SD	*p* Value
Mean age (years)	51.4 ± 18.6	47.7 ± 17.9	0.53 ^c^
Age range (years)	22–79	22–71	
Female gender, N (%)	18 (85%)	16 (84%)	0.72 ^b^
Dry eye classification, N (%)			0.26 ^d^
Sjögren’s syndrome	15 (57.9%)	11 (57.9%)	
Aqueous tear deficiency	3 (12%)	5 (26%)	
MGD	3 (12%)	3 (15.8%)	
Treated eye RE, N (%)	57.9%	68%	0.76 ^b^
IOP at baseline (mmHg)	14.5 ± 1.4	13.76 ± 0.9	0.31 ^a^
BCVA at baseline (LogMAR)	0.7 ± 0.1	0.7 ± 0.10	0.66 ^a^
Schirmer’s test at baseline (mm)	4.5 ± 1.5	5.2 ± 1.6	0.69 ^a^

^a^ Analyzed with Mann–Whitney test; ^b^ Chi-square; ^c^ *t*-test; ^d^ Fisher–Freeman–Halton exact test; BCVA—best corrected visual acuity; LogMAR—Logarithm of the Minimum Angle of Resolution; IOP—intraocular pressure; MGD—Meibomian gland dysfunction; RE—right eye; SD—standard deviation.

**Table 2:** Safety of repetitive magnetic stimulation.

Treatment	Measurement	Time	Mean ± SD	*p* Value (Change over Time)
Treated N = 21	BCVA	Enrollment	0.22 ± 0.28	0.13 ^a^
		1 week	0.17 ± 0.26	
		1 month	0.15 ± 0.26	
		2 months	0.15 ± 0.26	
		3 months	0.19 ± 0.26	
	IOP	Enrollment	13.76 ± 2.57	0.16 ^a^
		1 week	12.40 ± 2.68	
		1 month	11.96 ± 3.43	
		2 months	11.96 ± 2.65	
		3 months	12.90 ± 3.13	
	Schirmer	Enrollment	5.24 ± 4.32	0.42 ^b^
		1 week	4.44 ± 3.65	
		1 month	4.64 ± 4.00	
		2 months	4.13 ± 3.70	
		3 months	3.62 ± 2.71	
Not Treated N = 19	BCVA	Enrollment	0.14 ± 0.27	0.39 ^a^
		1 week	0.15 ± 0.28	
		1 month	0.14 ± 0.26	
		2 months	0.14 ± 0.26	
		3 months	0.16 ± 0.28	
	IOP	Enrollment	14.53 ± 3.24	0.74 ^a^
		1 week	13.05 ± 3.31	
		1 month	13.84 ± 3.43	
		2 months	13.42 ± 3.31	
		3 months	13.89 ± 3.13	
	Schirmer	Enrollment	4.53 ± 3.65	0.51 ^b^
		1 week	3.89 ± 2.76	
		1 month	3.47 ± 2.95	
		2 months	4.5 ± 4.1	
		3 months	4.47 ± 3.60	

^a^ Calculated using the Freidman test; ^b^ calculated using Greenhouse–Geisser test; BCVA = best corrected visual acuity; IOP = intraocular pressure.

**Table 3:** Comparative analysis of NEI and TBUT scores in treatment groups over time.

Time Point	Group	NEI Mean ± SD	TBUT Mean Rank ± SD	NEI *p* Value	TBUT *p* Value
Enrollment	Non-treated N = 19	6.68 ± 3.65	3.24 ± 2.72	0.52 ^a^	0.19 ^b^
Week 1		6.32 ± 3.71	3.64 ± 2.03		
Month 1		6.05 ± 3.29	2.7 ± 1.71		
Month 2		6.16 ± 3.16	4.22 ± 2.28		
Month 3		5.47 ± 3.85	3.99 ± 2.06		
Enrollment	Treated N = 21	9.76 ± 3.80	2.94 ± 1.56	0.004 ^a^	0.04 ^b^
Week 1		7.62 ± 4.43	3.26 ± 1.19		
Month 1		6.67 ± 4.35	3.19 ± 1.29		
Month 2		5.19 ± 3.60	3.77 ± 0.99		
Month 3		7.24 ± 5.02	3.99 ± 1.24		

^a^ Calculated using the Greenhouse–Geisser test; ^b^ calculated using the Friedman test; NEI—National Eye Institute; SD—standard deviation; TBUT—tear break-up time.

**Table 4:** Change in subjective assessment score over time by treatment group.

Treatment		Score Mean ± SD	*p* Value ^a^
Group	Time		
Non-treated	Enrollment score	12.50 ± 1.55	0.008
	1-week score	8.27 ± 1.36	
	1-month score	8.09 ± 1.62	
	2-month score	7.27 ± 1.61	
	3-month score	8.45 ± 1.45	
OCD	Enrollment score	7.42 ± 1.26	0.007
	1-week score	5.95 ± 1.07	
	1-month score	5.65 ± 1.23	
	2-month score	6.65 ± 1.38	
	3-month score	5.81 ± 1.45	

^a^ Calculated using the Friedman test; SD—standard deviation.

## Data Availability

The data set generated during the current study is available from the corresponding authors on reasonable request.

## Supplementary Materials

The following supporting information can be downloaded at https://www.mdpi.com/article/10.3390/biomedicines13051064/s1, Figure S1: The VIVEYE—Ocular Magnetic Neurostimulation System Ver 1.0—Device Overview; Figure S2: Study Visit Scheme.
