# Supplementary material for: Evaluating Magnetic Stimulation as an Innovative Approach for Treating Dry Eye Disease: An Initial Safety and Efficacy Study
Source: Biomedicines. 2025 Apr 28;13(5):1064. doi: 10.3390/biomedicines13051064 (PMC12109051; doi:10.3390/biomedicines13051064)

Supplementary Material:

Figure S1: The VIVEYE - Ocular Magnetic Neurostimulation System Ver 1.0 - Device Overview

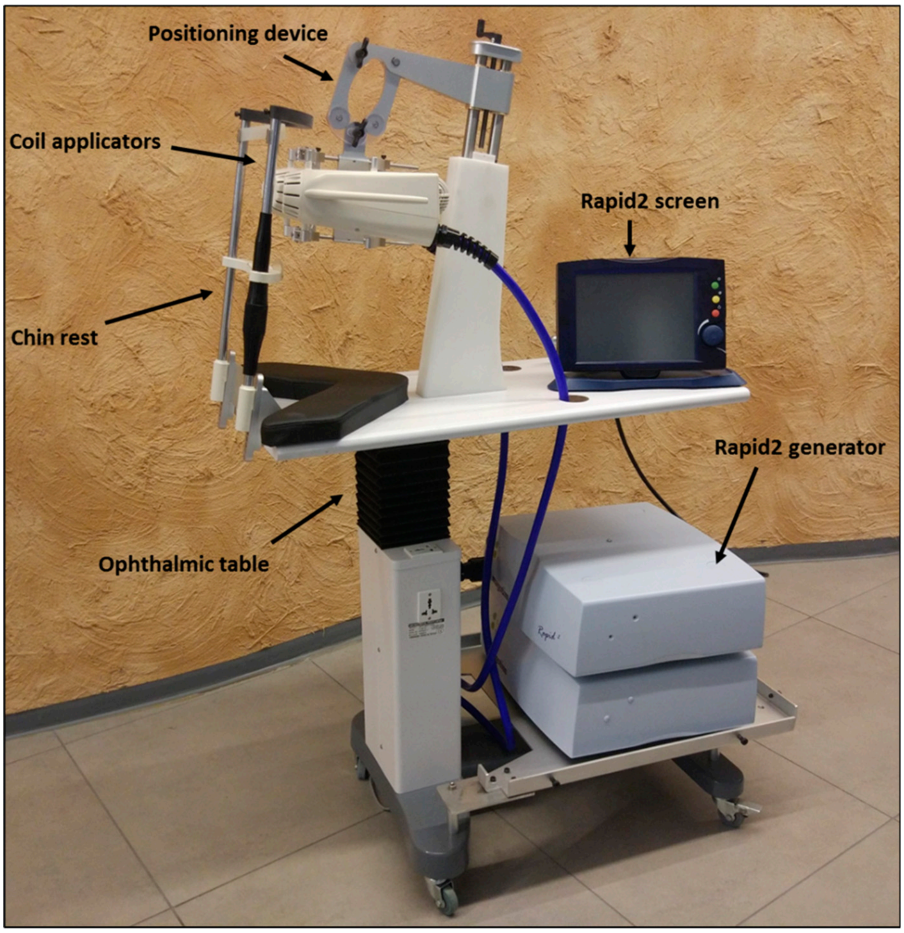

Supplementary Material:

Figure S2: Study Visit Scheme

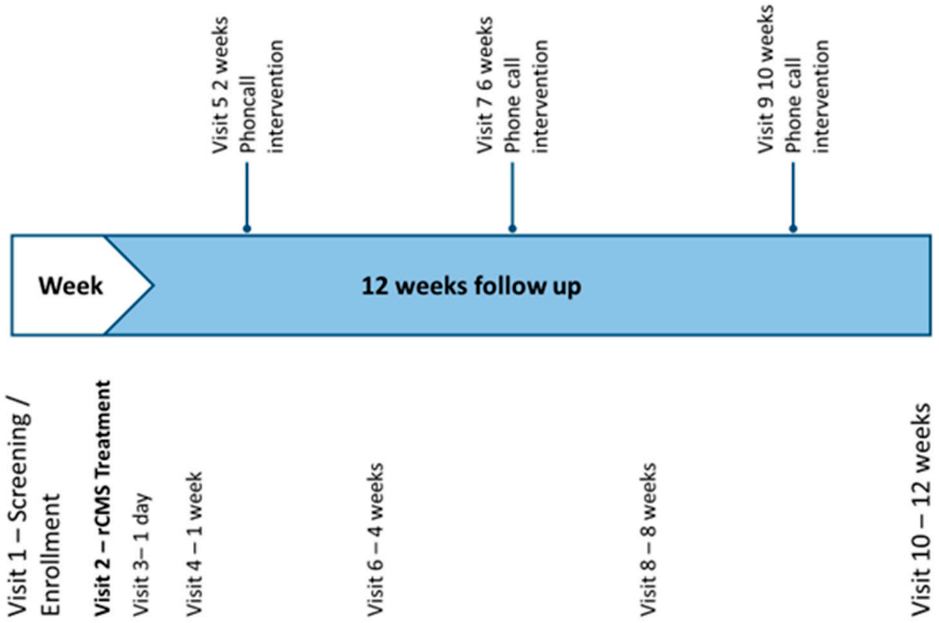

Supplement: Supplementary file 1 [file biomedicines-13-01064-s001.zip › biomedicines-3533390-supplementary.pdf]
